# A Green Extraction Method to Achieve the Highest Yield of Limonin and Hesperidin from Lime Peel Powder (*Citrus aurantifolia*)

**DOI:** 10.3390/molecules27030820

**Published:** 2022-01-26

**Authors:** Pakkapong Phucharoenrak, Chawanphat Muangnoi, Dunyaporn Trachootham

**Affiliations:** 1 Master of Science Program in Toxicology and Nutrition for Food Safety, Institute of Nutrition, Mahidol University, Nakhon Pathom 73170, Thailand; pakkapong.ruk@student.mahidol.edu; 2 Institute of Nutrition, Mahidol University, Nakhon Pathom 73170, Thailand; Chawanphat.mua@mahidol.ac.th

**Keywords:** food waste, citrus, lime, green extraction, limonin, hesperidin, LC-MS/MS

## Abstract

Green extraction is aimed at reducing energy consumption by using renewable plant sources and environmentally friendly bio-solvents. Lime (*Citrus aurantifolia*) is a rich source of flavonoids (e.g., hesperidin) and limonoids (e.g., limonin). Manufacturing of lime products (e.g., lime juice) yields a considerable amount of lime peel as food waste that should be comprehensively exploited. The aim of this study was to develop a green and simple extraction method to acquire the highest yield of both limonin and hesperidin from the lime peel. The study method included ethanolic-aqueous extraction and variable factors, i.e., ethanol concentrations, pH values of solvent, and extraction temperature. The response surface methodology was used to optimize extraction conditions. The concentrations of limonin and hesperidin were determined by using UHPLC-MS/MS. Results showed that the yields of limonin and hesperidin significantly depended on ethanol concentrations and extraction temperature, while pH value had the least effect. The optimal extraction condition with the highest amounts of limonin and hesperidin was 80% ethanol at pH 7, 50 °C, which yields 2.072 and 3.353 mg/g of limonin and hesperidin, respectively. This study illustrates a green extraction process using food waste, e.g., lime peel, as an energy-saving source and ethanol as a bio-solvent to achieve the highest amount of double bioactive compounds.

## 1. Introduction

Plant extract is one of the main ingredients in functional food, dietary supplements and herbal medicine. Production of plant extract usually requires growing a large amount of plant as raw materials and utilizing toxic solvents for extraction. Thus, a green extraction concept has been proposed as a desirable approach to save energy, reduce environmental problems, and promote sustainable development [1]. Chemat et al. proposed that “green extraction is based on the discovery and design of extraction processes which will reduce energy consumption, allow the use of alternative solvents and renewable natural products, and ensure a safe and high-quality extract/product” [1]. To obtain limitless and energy-saving plant sources for extraction, clean waste from the food industry can be a good candidate.

Lime (*Citrus aurantifolia*) is a citrus fruit that has been cultivated around the world. Its annual world production is approximately 20,050,000 metric tons [2]. The fruit is an industrial drop that is sold in both fresh markets and the food industry. Nevertheless, the commonly consumed part of the lime is the juice, which accounts for about 48% of the total weight, while the rest (peel, seed, and dehydrated flesh: 52% of total weight) is usually unused, thus becoming food waste [3]. To promote responsible consumption and production, according to the sustainable development goals, such a significant amount of food waste from lime use should be managed and comprehensively exploited. Utilizing food waste such as the lime peel not only reduces environmental problems but also adds commercial value to limes [4].

Previous studies have shown that lime is a rich source of phytochemical compounds, especially flavonoid and limonoid groups. These compounds can be found in all parts of the lime, mostly abundant in the outer and inner layers of the peel (flavedo and albedo) [3,5,6]. The prominent phytochemical compounds that are reported to be found in waste products from lime are limonin and hesperidin. Limonin is a natural tetracyclic triterpenoid compound in the limonoid group. It is a secondary metabolite that is widely found in citrus fruits [7]. Numerous studies have shown that limonin exhibits a wide spectrum of biological and pharmacological activities, including anti-cancer [8,9,10], anti-inflammatory [10,11], anti-oxidative [12], anti-viral [13], and liver-protective properties [14,15]. Hesperidin, or vitamin P, is a flavanone glycoside found in all types of citrus fruits. It is one of the safest and most valuable bioactive compounds, which possess a wide range of pharmacological properties [16]. Hesperidin has exhibited anti-type II diabetic [16], anti-inflammatory [17], anti-oxidative [17,18], anti-cancer [19,20], and cardioprotective properties [21,22], etc. These findings suggest that limonin and hesperidin can provide health-promoting benefits. Nevertheless, the traditional extraction methods for limonin and hesperidin usually require organic solvents that are toxic and harmful to the environment (e.g., dichloromethane, ethyl acetate, toluene, etc.) [23,24]. New extraction methods such as supercritical carbon dioxide (SC-CO_2_) have been developed to solve this problem [25]. SC-CO_2_ extraction reduces the organic solvent for extraction, resulting in a more environmentally friendly process. Nevertheless, this process requires high costs, sophisticated devices, and special expertise [24,25]. Thus, alternative extraction methods that use energy-saving plant sources and environmentally friendly bio-solvents are necessary. The present study is aimed at developing a green and simple extraction method to acquire the highest yield of both limonin and hesperidin from lime peel. Factors affecting extraction efficiency were studied, and the optimum green extraction method was proposed to maximize the use of food waste for appreciable cost-effectiveness and environmental sustainability.

## 2. Results

### 2.1. Chromatograms and Mass Spectra of Limonin and Hesperidin

**Figure 1:**
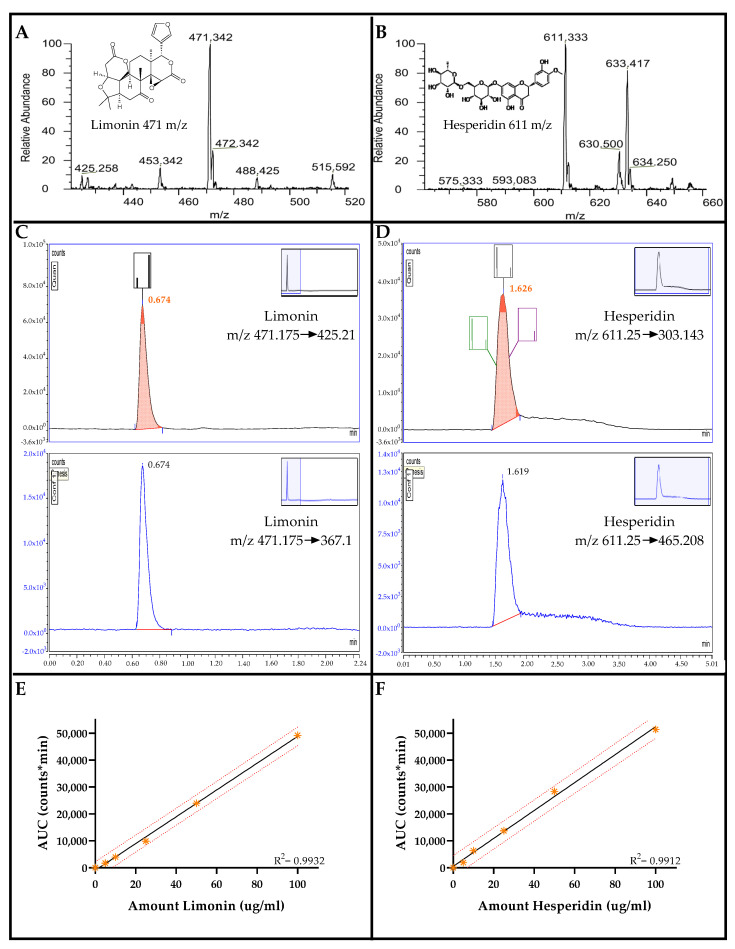
Reference compounds analyzed by UHPLC-MS/MS. Precursor MS spectra of limonin (**A**) and hesperidin (**B**). Chromatograms for quantified (upper) and confirmed (lower) product ions of limonin (**C**) and hesperidin (**D**). Retention times are shown as a red number above the quantified peaks. Average calibration curves of limonin (**E**) and hesperidin (**F**) generated by the linear plots between areas under the curves of the quantitative product ions and the concentrations of standard solutions. R^2^ values were obtained from linear regression.

Chromatogram and mass spectra of limonin and hesperidin obtained from ultra-high performance liquid chromatography and tandem mass spectrometry (UHPLC-MS/MS) are shown in Figure 1. The precursor mass of limonin was *m/z* 471 (Figure 1A). The quantified and confirmed product ions of limonin were *m/z* 425 and 367, respectively (Figure 1B). The precursor mass of hesperidin was *m/z* 611 (Figure 1C). The quantified and confirmed product ions of limonin were *m/z* 303 and 465, respectively (Figure 1D). The retention times of limonin and hesperidin were 0.6 and 1.6 min, respectively. Calibration curves showed good linearity with an average R^2^ of 0.9932 and 0.9912 for limonin and hesperidin, respectively (Figure 1E,F). Intra-day precision for limonin and hesperidin was 0.26–8.33% and 0.76–7.76% RSD, respectively. Inter-day precision for the analyses of limonin and hesperidin was 0.43–9.58 and 0.46–10.75, respectively.

### 2.2. Effect of Extraction Factors on Limonin and Hesperidin Concentration

**Figure 2:**
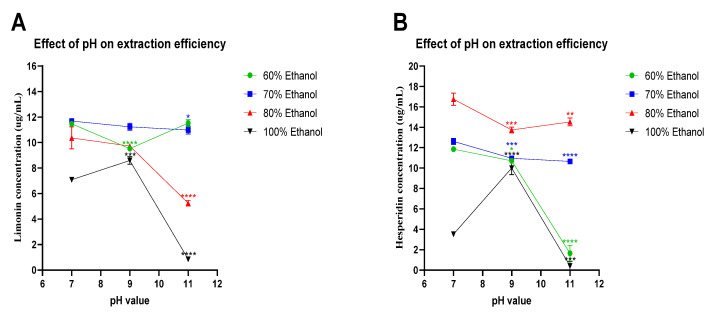
Effect of pH on extraction efficiency. Limonin (**A**) and hesperidin (**B**) concentrations in the ethanolic-aqueous extracts of lime peel after extraction with 60% (●), 70% (■), 80% (▲), and 100% (▼) ethanol in water with the specified pH values at 50 °C for 100 min. The statistical differences were separately analyzed for limonin and hesperidin by using one-way ANOVA followed by Tukey’s multiple comparisons test. * , **, *** and **** mean *p* < 0.05, <0.01, <0.001 and <0.0001, respectively, compared with the concentrations of limonin or hesperidin at pH 7 of each specified condition.

**Figure 3:**
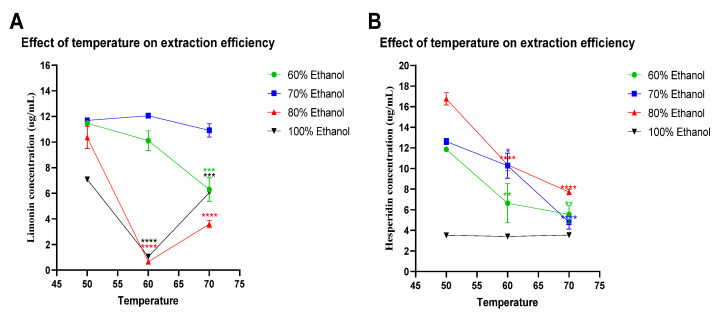
Effect of temperature on extraction efficiency. Limonin (**A**) and hesperidin (**B**) concentrations in the ethanolic-aqueous extracts of lime peel after extraction with 60% (●), 70% (■), 80% (▲), and 100% (▼) ethanol in water with the specified temperatures, at pH 7 for 100 min. The statistical differences were separately analyzed for limonin and hesperidin by using one-way ANOVA followed by Tukey’s multiple comparisons test. * , **, *** and **** mean *p* < 0.05, <0.01, <0.001 and <0.0001, respectively, compared with the concentrations of limonin or hesperidin at 50 °C of each specified condition.

As shown in Figure 2, the yields of limonin and hesperidin concentrations were varied upon ethanol concentrations and pH values. At pH 7 and 50 °C, extraction with 80% ethanol showed the highest yields among all ethanol concentrations tested. However, pH values of ethanol solutions also affected limonin and hesperidin concentration. The concentration of limonin and hesperidin tended to decrease when the pH value increased from 7 to 9 and from 7 to 11, respectively, for most ethanol concentrations, except for those of 100% ethanol at pH 9. Furthermore, temperature is also another factor affecting extraction efficiency. As shown in Figure 3, at pH 7 when the extraction temperature rises from 50 to 60 °C and from 50 to 70 °C, respectively, the yields of limonin and hesperidin concentrations seemed to decrease in most conditions, except for those of hesperidin after extraction with 100% ethanol (no changes upon temperature rise).

As shown in Figure 2 and Figure 3, regardless of pH or temperature, the use of mixed solvents (ethanol and DI water) provided higher yields of limonin and hesperidin than those of the pure solvent (100% ethanol). The highest concentrations of limonin and hesperidin were achieved by extraction with 70% and 80% ethanol (*v/v*), respectively.

### 2.3. Optimization of Extraction Conditions

**Figure 4:**
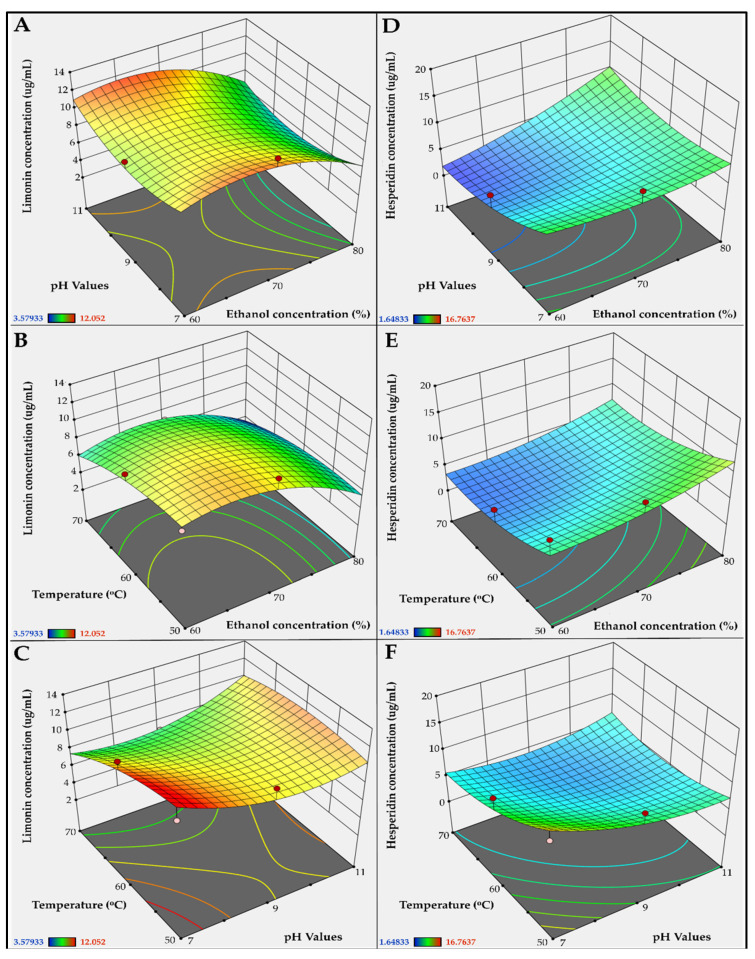
Relationship between extraction factors. Three-dimensional (3D) response surface methodology (RSM) diagrams show concentrations of limonin (**A**–**C**) and hesperidin (**D**–**F**) in the ethanolic-aqueous extracts of lime peel as the function of ethanol concentration, extraction temperature, and pH value of extraction solvent. Red, yellow, green and blues areas depict the highest to lowest concentrations of limonin and hesperidin.

The results of this study showed that ethanol concentration, pH value of solvent, and extraction temperature were key factors affecting the yields of limonin and hesperidin. When analyzed with ANOVA, the interactions between these factors were found. Therefore, response surface methodology (RSM) was used to demonstrate the relationship between these factors and was used to identify the most influential factor. Figure 4A–C and Figure 4D–F show the RSM models for limonin and hesperidin concentration, respectively. The optimum values of the factors are shown within the warm tone color (maximum within red) regions. The response surface analysis (RSA) using ANOVA showed that the extraction yield of limonin depended most on ethanol concentration, followed by extraction temperature. The extraction yield of hesperidin depended most on extraction temperature followed by ethanol concentration. The pH value had the least effect on the extraction yields of both limonin and hesperidin.

### 2.4. Yields of Limonin and Hesperidin in Ethanolic-Aqueous Extracts of Lime Peel

The extraction yield was calculated as mg of limonin or hesperidin per gram of dry peel. As shown in Table 1, the condition yielding the highest amount of limonin was the extraction with 70% ethanol at pH 7 and 60 °C, while the condition yielding the highest amount of hesperidin was the extraction with 80% ethanol at pH 7 and 50 °C. For limonin, the extraction yields of 70% and 80% ethanol were not significantly different. However, for hesperidin, 80% ethanol provided a significantly better yield. Therefore, the optimal extraction condition achieving the highest amounts of limonin and hesperidin was 80% ethanol at pH 7, 50 °C.

## 3. Discussion

A number of scientific evidence supports the use of plant extracts to promote good health and wellbeing [26]. However, traditional methods for plant extraction are energy-consuming and environmentally unfriendly [1]. In this study, we demonstrated a good example of green extraction by utilizing food waste such as lime peel as a plant source and by using ethanol–water as a bio-solvent to achieve the highest amount of double bioactive compounds, i.e., limonin and hesperidin. LC-MS/MS was used to accurately determine the amount of bioactive compounds in the extract. The findings of this work suggest that ethanol concentration and extraction temperature were key factors affecting the extraction yields of limonin and hesperidin, while pH value had the least effect. The optimal extraction condition achieving the highest amounts of limonin and hesperidin was 80% ethanol at pH 7, 50 °C, which yields 2.072 and 3.353 mg/g of limonin and hesperidin, respectively.

Previous studies mostly used toxic organic solvents such as dimethyl sulfoxide (DMSO), dichloromethane, ethyl acetate, and toluene for plant extraction of limonin and hesperidin [23,24]. Instead, less toxic solvents, i.e., ethanol and water, were chosen for extraction in this study. In comparison with previous studies [12,27], the extraction yields of limonin and hesperidin in this work are relatively lower. The yield ranges for limonin and hesperidin in previous studies were 2.3–11.38 and 0.0–3.6 mg/g, respectively. The yield ranges for limonin and hesperidin in this study were 0.170–2.191 and 0.003–3.353 mg/g. Furthermore, the effect of pH on extraction yields, which was shown to be crucial in previous studies, plays a minor role according to our current study. One of the main reasons of the discrepancy is the difference in detection methods. Previous works mostly used high-performance liquid chromatography (HPLC) to detect limonin and hesperidin in plant products, including in citrus fruits [28]. However, the limitation of HPLC is its inability to separate compounds that have similar properties in interactions of the mobile and stationary phases [28,29,30]. Analysis of bioactive compounds using HPLC usually produced high yields, but they were not entirely specific. Therefore, HPLC mostly provides a rough estimate of the whole class of compounds (e.g., limonoids or flavanone) rather than the accurate determination of specific bioactive compounds (e.g., limonin and hesperidin). In contrast, liquid chromatography–tandem mass spectrometry (LC-MS/MS) can determine the existence and the amount of specific compounds based on mass-to-charge ratio of ions and fragment ions that are produced during the ionization process [31]. In comparison with HPLC, LC-MS/MS provides a more accurate separation and quantification of specific bioactive compounds. Therefore, in this study, LC-MS/MS was chosen to specifically determine the amounts of limonin and hesperidin.

In general, the efficiency of plant extraction mainly depends on type of solvent, duration, pH value, and temperature [32]. Therefore, in this study, we investigated the impact of those factors. In the case of limonin, previous studies have reported that pH value had a significant effect on limonin concentration through the activity of Limonin-D-ring-lactonase (LDRLase) [24]. LDRLase is an enzyme found in citrus fruits that catalyzes the reversible conversion between limonin and limonoate A-ring lactone (LARL). The activity of LDRLase depends on pH value. In an alkaline condition (pH ≥ 8), LDRLase catalyzes a ring-opening reaction through hydrolysis of the D-ring in limonin and converts it into LARL [33]. LC-MS/MS is specific to limonin; therefore, the LARL form will not be detected at the same measurement. This fact may explain why the yield of limonin decreases when extracted at a higher pH. Nevertheless, according to our response surface methodology, pH value of extraction solvent had the smallest effect on limonin concentration, compared to ethanol concentration and extraction temperature. Likewise, pH value of extraction solvent had the least effect on hesperidin concentration. Previous studies have shown that the solubility of hesperidin significantly increased at higher pH. However, at a pH value greater than 9, degradation of hesperidin could also occur [34]. Thus, increasing the pH did not result in a significantly higher hesperidin concentration, when hesperidin was specifically determined by LC-MS/MS.

In this study, the extraction temperature and ethanol concentration were shown to play an important role in the extraction efficiency of limonin and hesperidin from lime peel. For ethanol concentration, we found that the use of mixed solvents (ethanol and DI water) provided higher yields of limonin and hesperidin than those of pure ethanol. Consistently, a previous report by Gertenbach demonstrated that the presence of water in the solvent mixture causes the plant cells to swell, allowing easier diffusion of the solvent into the plant cells [35]. For extraction temperature, we found that an increase in the extraction temperature to higher than 50 °C actually reduced the yield of limonin and hesperidin. Generally, the solubility of bioactive compounds is increased with a rise in temperature; thus, heat is expected to increase extraction efficiency [36]. Nevertheless, the effect of temperature on extraction also depends on the chemical properties of the extracted compounds. In the case of limonin and hesperidin, previous studies reported that, as the temperature rises, limonin can significantly degrade [37], and hesperidin can be converted into its aglycone form (hesperetin) through chemical or enzymatic processes [34]. It was also reported that hesperidin glycosylated form and hesperidin complex has high thermal stability, but pure hesperidin has low thermal stability. These structural differences cannot be detected with the HPLC technique [38], but they can be distinguished with LC-MS/MS. In this study, we used LC-MS/MS to specifically determine only limonin and hesperidin. Therefore, temperature-dependent degradation or conversion can result in a decrease in limonin concentration when detected with LC-MS/MS. Future studies using LC-MS/MS are warranted to characterize various forms of limonin and hesperidin derivatives in varied extraction conditions. However, this study indicated that the pH values of extracts could be different from those of the solvents, which is consistent with a previous study by Hosseini that extracted from red cabbage, barberry, and eggplant peel. They reported that pH values of the extract may change after extraction and were found to differ when extracted with different solvents [39]. This may be the result of various extraction factors, such as types and percentage of the extraction solvent, properties of the sample, solid-to-solvent ratio, etc., that affect the extraction of acidic substances in the sample. Future investigation of this point is worthwhile.

Considering the interaction between several factors affecting extraction efficiency, our response surface methodology data is consistent with previous studies. Qin et al. showed that extraction temperature had a profound effect on the limonin concentration in the extract of pummelo seeds [12]. Another study, which extracted hesperidin from three types of citrus peels (orange, lemon, and clementine), found that ethanol concentration significantly affected hesperidin concentration in every sample, while the extraction temperature had a significant effect on some samples [40]. The most influential factors on extraction efficiency may vary depending on the species or part of the plant used for extraction. Furthermore, other factors, e.g., solid-to-solvent ratio, time for extraction, level of shaking, etc., also impact extraction yields. Therefore, to obtain consistently high yields of plant extract, optimization of the extraction method and good quality control of the extraction condition are absolutely required.

The strength of this work includes the detailed design of varied conditions for ethanol concentration, temperature, and pH to elucidate the interactions among these extraction factors. In addition, the use of LC-MS/MS allows for accurate and specific measurements of the bioactive compounds. Nevertheless, the limitations of this work include the fact that only temperatures of 50 °C and above and pH of 7 and above were tested. Furthermore, the lime peel powder used for extraction was randomly collected from the same batch of the industrial food waste. Future studies are warranted to compare the extraction yields of limonin and hesperidin from various batches of food waste. Calculation of the coefficient of variations (CV) is required to determine the consistency. Using industrial food waste, such as lime peel, from a standardized lime juice factory not only saves energy in growing new lime trees, but it also benefits from the good manufacturing practice (GMP) system to control the consistency of raw materials, i.e., the source of lime. Another limitation of this study is that we only used ethanol as an extraction solvent. The aim of this work was to develop a green extraction process. Therefore, ethanol was chosen based on its low cost, solubility, and environmentally friendly properties. This study used ethanol for extraction and LC-MS-MS for quantitation of extraction yields, while previous studies used more harmful solvents for extraction and HPLC for analysis. Therefore, the extraction yields obtained in this study cannot be fairly compared with previous works. Future studies are warranted to compare the extraction yields of ethanol with other types of solvents in parallel experiments and using similar analytical methods such as LC-MS/MS.

This study discovered an optimized green extraction method to achieve the highest yields of limonin and hesperidin by using 80% ethanol in water to extract lime peel at pH 7 and 50 °C. The limonin–hesperidin-rich ethanolic-aqueous extracts of lime peel are expected to be highly effective owing to the dual bioactive compounds. Future in vitro and in vivo studies are warranted to investigate its health benefits such as anti-cancer, anti-inflammatory and anti-oxidative properties.

## 4. Materials and Methods

### 4.1. Chemicals and Raw Materials

Lime peel powder was obtained from Chiangmai Bioveggie Co., Ltd. (Chiang Mai, Thailand). The powder was produced by vacuum drying fresh lime peel, which is the food waste from the GMP-certified lime juice factory. Analytical standard of limonin (≥95.0% purity) and hesperidin (≥97.0% purity) were purchased from Supelco, Inc. (Bellefonte, PA, USA). HPLC-grade ethanol (≥99.8% purity), sodium hydroxide (≥98% purity), citric acid (≥99.5% purity) was purchased from Sigma-Aldrich (St. Louis, MO, USA). Phosphate-buffered saline (pH 7.4) was purchased from Invitrogen Life Science Technologies, Thermo Fisher Scientific, Inc. (Waltham, MA, USA).

### 4.2. Experimental Design

Based on the chemical properties of limonin [41] and hesperidin [34], three variables, namely solvent dosage, pH of solvent, and temperature of extraction, were selected to vary the extraction conditions. Other factors, including time for extraction, the level of shaker and solid-to-solvent ratio, remained constant in all experiments. Ethanol was chosen as the extraction solvent in this study because it was a relatively low-cost organic solvent that classified as an environmentally friendly preferable green solvent and commonly used in various industries [42]. Lime peel powder was extracted with 60%, 70%, 80% ethanol in water, and 100% ethanol, and varied pH values of solvent at 7, 9, and 11. All extraction experiments were performed with a solid-to-solvent ratio of 0.01 g/mL in a shaking water bath (Memmert GmBH Co., Büchenbach, KG, Germany) at shaking speed level of 3.5 for 100 min. The extraction temperatures varied at 50, 60, or 70 °C. All conditions of extraction were performed three times separately, and three independent samples were used for data analysis. Response surface methodology was used to determine the optimum extraction conditions to extract limonin and hesperidin by varying operating parameters according to 3 × 3 factorials. Then, the concentrations of limonin and hesperidin were measured by UHPLC-MS/MS.

### 4.3. UHPLC-MS/MS Analysis

Measurement of limonin and hesperidin was performed with a method adapted from a previous study [43,44]. The extracts were collected and filtered through a 0.2 micron nylon syringe filter. The extracts were adjusted pH values to 7.4 with sodium hydroxide or citric acid solution. The final volumes were adjusted by using phosphate-buffered saline (pH 7.4) prior to analysis. The samples were analyzed with ultra-high performance liquid chromatography (UHPLC)-tandem mass spectrometry by using Ultimate 3000 connected with a TSQ Quantis Triple Quadrupole Mass Spectrometer (Thermo Scientific, Waltham, MA, USA). UHPLC was performed by using Hypersil GOLD C18 column (1.9 μm particle size, 100 mm × 2.1 mm, ThermoScientific, Waltham, MA, USA). The column was maintained at 40 °C. Gradient run at a flow rate of 0.3 mL/ min for 5.10 min was performed using two types of mobile phase, i.e., 0.1% formic acid in deionized water (A) and 0.1% formic acid in methanol (B). The gradient program included: 0–1.3 min, 15–30% B; 1.30–2.50 min, 30–60% B; 2.50–5.10 min, 60–60% B. The injection volume was 1 µL. Tandem mass spectrometric analysis was performed in electron spray ionization with positive ion mode (ESI+) at spray voltage of 3.5 kV, ion transfer tube temperature at 350 °C, vaporizer temperature at 400 °C, sheath gas, auxiliary gas and sweep gas at 60, 15 and 2 arbitrary units, respectively. Mass spectrometer analyses of all samples were performed in selected reaction monitoring (SRM) mode for simultaneous analysis of multiple masses. The mass-to-charge ratio (*m/z*) of limonin precursor and quantified product mass were 471 and 425, respectively. The collision energy of 20 V was used for the transition. The confirmation product mass for limonin was *m/z* 367 with the collision energy of 19 V for the transition. For hesperidin, the precursor and quantified product mass were *m/z* 611 and 303, respectively. The collision energy of 20 V was used for the transition. The confirmation product mass for hesperidin was *m/z* 465 with the collision energy of 12 V for the transition.

Standard curves were generated from standard solution of single compound (limonin or hesperidin) at the concentrations of 5, 10, 25, 50, 100 µg/ mL in ethanol. The areas under the curve (AUC) of MS chromatograms for the quantified product mass and the corresponding concentrations of limonin and hesperidin were used to create linear standard curves.

### 4.4. Statistical Analysis

Statistical analyses were performed using PASW Statistics 18 (formerly SPSS Statistics) or GraphPad prism V.9. Data were expressed as means ± SD of at least 3 independent experiments (three separate extractions). One-way analysis of variance (ANOVA) with Tukey’s multiple comparisons or Ryan–Einot–Gabriel–Welsh F (REGWF) post hoc test was performed to compare the mean of each experimental condition. Multi-factorial ANOVA was used to determine the interaction effect of independent variables. A *p* value < 0.05 indicated a statistically significant difference.

## 5. Conclusions

This study demonstrated that lime peel obtained as food waste from a lime juice factory could be a potential renewable plant source for limonin and hesperidin—a rich extract. Using LC-MS/MS for determination of the bioactive compounds together with response surface methodology, we discovered that solvent concentration and extraction temperature were crucial factors that influenced extraction yields. The optimum condition to obtain the highest yields of limonin and hesperidin from lime peel was extraction with 80% ethanol at a solid-to-solvent ratio of 0.01 g/mL and pH 7 at 50 °C for 100 min. This condition provided the yields of limonin and hesperidin at 2.072 and 3.353 mg/g, respectively. The method used ethanol and water as the extraction solvent, which is safer for extractors and more environmentally friendly than other previously used organic solvents. Therefore, this process offers a good alternative that may be applied for implementation on an industrial scale as a green extraction process of limonin and hesperidin.

## Figures and Tables

**Table 1:** Yields of limonin and hesperidin in lime peel ethanolic-aqueous extracts; each extraction condition is at a solid-to-solvent ratio (SSR) of 0.01 g/mL.

Extraction Condition			pH of Extract (before Adjusting)	Limonin Yield (mg/g Dry Peel)	Hesperidin Yield (mg/g Dry Peel)
%ETOH	pH	Temp.			
60	7	50	4.15	2.086 ± 0.015 ^abc^	2.373 ± 0.023 ^ef^
60	7	60	4.12	1.838 ± 0.140 ^fghi^	1.327 ± 0.379 ^hi^
60	7	70	4.12	1.143 ± 0.167 ^nop^	1.108 ± 0.172 ^ijk^
60	9	50	3.98	1.731 ± 0.022 ^ij^	2.142 ± 0.015 ^fg^
60	9	60	4.08	1.749 ± 0.033 ^hij^	1.196 ± 0.023 ^ij^
60	9	70	4.01	1.540 ± 0.011 ^lm^	0.605 ± 0.011 ^nop^
60	11	50	4.25	2.095 ± 0.051 ^abc^	0.330 ± 0.153 ^qrs^
60	11	60	4.43	1.548 ± 0.031 ^lm^	0.876 ± 0.010 ^klmn^
60	11	70	4.20	1.577 ± 0.025 ^klm^	0.665 ± 0.010 ^mnop^
70	7	50	4.22	2.125 ± 0.036 ^ab^	2.523 ± 0.058 ^de^
70	7	60	4.27	2.191 ± 0.024 ^a^	2.054 ± 0.243 ^g^
70	7	70	4.29	1.984 ± 0.094 ^bcdef^	0.958 ± 0.135 ^jkl^
70	9	50	4.29	2.043 ± 0.049 ^abcd^	2.191 ± 0.027 ^fg^
70	9	60	4.28	1.796 ± 0.053 ^ghi^	1.230 ± 0.085 ^ij^
70	9	70	4.43	1.264 ± 0.002 ^n^	0.588 ± 0.026 ^opq^
70	11	50	4.45	1.996 ± 0.056 ^bcde^	2.131 ± 0.032 ^fg^
70	11	60	4.46	1.909 ± 0.072 ^defgh^	0.801 ± 0.014 ^lmno^
70	11	70	4.49	1.260 ± 0.013 ^n^	0.590 ± 0.013 ^op^
**80**	**7**	**50**	**4.50**	**2.072 ± 0.173 ^abcd^**	**3.353 ± 0.121 ^a^**
80	7	60	4.46	0.653 ± 0.013 ^r^	0.370 ± 0.013 ^qr^
80	7	70	3.60	0.716 ± 0.058 ^r^	1.544 ± 0.055 ^h^
80	9	50	5.44	1.944 ± 0.019 ^cdefg^	2.748 ± 0.057 ^bcd^
80	9	60	4.63	0.894 ± 0.002 ^q^	0.824 ± 0.006 ^klmno^
80	9	70	3.64	1.537 ± 0.041 ^lm^	2.707 ± 0.111 ^cd^
80	11	50	8.61	1.048 ± 0.046 ^p^	2.906 ± 0.078 ^bc^
80	11	60	10.48	0.173 ± 0.004 ^s^	0.770 ± 0.011 ^lmno^
80	11	70	9.42	1.607 ± 0.026 ^jkl^	3.003 ± 0.038 ^b^
100	7	50	5.03	1.417 ± 0.376 ^m^	0.705 ± 0.041 ^lmno^
100	7	60	5.10	1.052 ± 0.066 ^op^	0.681 ± 0.048 ^lmno^
100	7	70	4.66	1.208 ± 0.025 ^no^	0.708 ± 0.037 ^lmno^
100	9	50	5.28	1.720 ± 0.059 ^ijk^	2.000 ± 0.130 ^g^
100	9	60	5.97	0.738 ± 0.056 ^r^	0.341 ± 0.007 ^qrs^
100	9	70	6.07	0.692 ± 0.027 ^r^	0.371 ± 0.004 ^pqr^
100	11	50	10.43	0.170 ± 0.004 ^s^	0.081 ± 0.084 ^st^
100	11	60	10.86	0.174 ± 0.003 ^s^	0.218 ± 0.109 ^rst^
100	11	70	10.84	0.173 ± 0.004 ^s^	0.003 + 0.001 ^t^

Table shows mean ± SD (*n* = 3). ^a–t^ Different superscript letters mean significant differences (*p* < 0.05) and same letter mean significant differences (*p* ≥ 0.05), obtained from ANOVA with Ryan–Einot–Gabriel–Welsh F post hoc test. Grey shade depicts highest yields.

## Sample Availability

Samples of the compounds are available from the authors upon a reasonable request.
